# CASC21, a FOXP1 induced long non-coding RNA, promotes colorectal cancer growth by regulating CDK6

**DOI:** 10.18632/aging.103376

**Published:** 2020-06-25

**Authors:** Tao Gong, Yu Li, Liang Feng, MingZhi Fang, Guoliang Dai, Xin Huang, Ye Yang, Shenlin Liu

**Affiliations:** 1 Oncology, Nanjing Hospital of Chinese Medicine, Nanjing Hospital of Chinese Medicine Affiliated to Nanjing University of Chinese Medicine, Nanjing 210000, Jiangsu, China; 2 Oncology, Jiangsu Province Hospital of Chinese Medicine, Affiliated Hospital of Nanjing University of Chinese Medicine, Nanjing 210000, Jiangsu, China; 3 Oncology, School of Traditional Chinese Pharmacy, China Pharmaceutical University, Nanjing 210000, Jiangsu, China; 4 Department of Clinical Pharmacology, Jiangsu Province Hospital of Chinese Medicine, Affiliated Hospital of Nanjing University of Chinese Medicine, Nanjing 210000, Jiangsu, China; 5 School of Medicine and Life Sciences, Nanjing University of Chinese Medicine, Nanjing 210000, Jiangsu, China

**Keywords:** CASC21, miR-539-5p, CDK6, colorectal cancer, lncRNA

## Abstract

Emerging studies indicate that long non-coding RNAs (lncRNAs) play crucial roles in colorectal cancer (CRC). Here, we reported lncRNA CASC21, which is induced by FOXP1, functions as an oncogene in CRC. We systematically elucidated its clinical significance and possible molecular mechanism in CRC. LncRNA expression in CRC was analyzed by RNA-sequencing data in TCGA. The expression level of CASC21 in tissues was determined by qRT-PCR. The functions of CASC21 was investigated by *in vitro* and *in vivo* assays (CCK8 assay, colony formation assay, EdU assay, xenograft model, flow cytometry assay, immunohistochemistry (IHC) and Western blot). Chromatin immunoprecipitation (ChIP), RNA immunoprecipitation (RIP) and luciferase reporter assays were utilized to demonstrate the potential mechanisms of CASC21. CASC21 is overexpressed in CRC and high CASC21 expression is associated with poor survival. Functional experiments revealed that CASC21 promotes CRC cell growth. Mechanistically, we found that CASC21 expressed predominantly in the cytoplasm. CASC21 could interact with miR-539-5p and regulate its target CDK6. Together, our study elucidated that CASC21 acted as an oncogene in CRC, which might serve as a novel target for CRC diagnosis and therapy.

## INTRODUCTION

CRC is one of the most common digestive system’s cancers. Its morbidity ranks third in all malignant tumors and it accounts for one-tenth of all tumor-related death worldwide [1, 2]. With the development of diagnostic and therapeutic techniques, the prognosis of CRC has improved significantly. However, for advanced CRC, there is still no effective treatment methods [3, 4]. It is necessary to further explore the molecular mechanism regulating the development and progression of CRC.

LncRNA is a type of non-coding RNA with a length greater than 200 nucleotides (nt) [5]. Most lncRNAs are structurally like mRNA, have a 5' end cap structure and a poly-A tail, and are transcribed from RNA polymerase II. They were originally considered to be ‘transcriptional noise’ [6, 7]. With the deepening of research, the functions of lncRNA are gradually recognized. Some lncRNAs were reported that can affect target genes expression at the transcriptional and translational levels and play important roles in many physiological and pathological processes [8, 9]. Recent studies have shown that several lncRNAs, such as CCAT1, CCAT2, H19 and HOTAIR, are abnormally expressed and play a vital role in CRC [10–13]. However, the roles of many other lncRNAs in CRC are not clear and further studies are needed.

Sustaining proliferation and evading growth suppressors are hallmarks of cancer [14]. Cancer cells always avoided normal controls of cell cycle progression which halt proliferation in the presence of physiological damages and had unscheduled proliferation abilities. This often mediated by alterations in cyclin-dependent kinase (CDK) activity [15, 16]. CDK6 is the catalytic subunit of the CDK6-cyclin D complex involved in the G1 to S cell cycle transition. Up-regulation of CDK6 is associated with the development and progression of many types of cancers [17, 18]. Recently, several lncRNAs have been reported to have a hand in the regulation of CDK6 expression. Yoshihiro et al. revealed that lncRNA MYU could interact with the RNA binding protein hnRNP-K to stabilize CDK6 expression and promote cell cycle progression of CRC cells [19]. Wang et al. demonstrated that Lnc-UCID increased CDK6 expression in hepatoma by competitively binding to DHX9 and sequestering DHX9 from CDK6-3’UTR [20]. Liu et al. reported that lncRNA HNF1A-AS1 could regulate CDK6 expression via sponging with miR-149-5p [21].

In the present study, we identified the abnormally expressed lncRNAs in CRC. We first reported that CASC21, a FOXP1 induced lncRNA, which can promote the development of CRC. We found that CASC21 was highly expressed in CRC cells and tissues and its high expression was associated with poor prognosis. It can increase CDK6 expression by sponging miR-539-5p and promote cell cycle transition of CRC cells. Our study provides a new theoretical basis for the diagnosis and treatment of CRC.

## RESULTS

### CASC21 is highly expressed in CRC and correlates with prognosis of CRC patients

**Figure 1:**
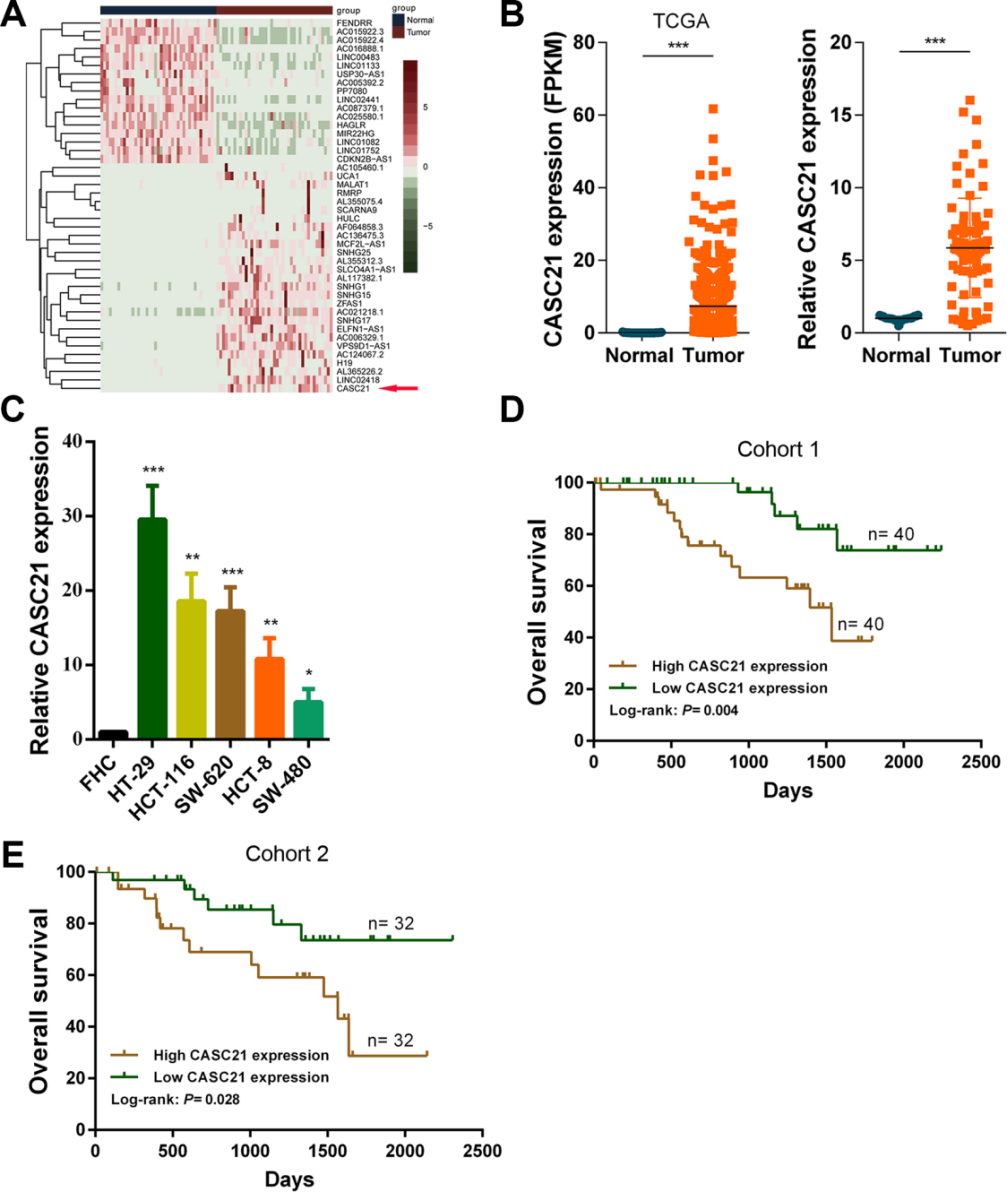
**CASC21 expression is elevated in CRC and high CASC21 expression predicts poor prognosis.** (**A**) Heatmap of abnormally expressed lncRNAs in CRC in TCGA database. Red in the heat map indicates upregulation, green indicates downregulation. The red arrow denotes CASC21. (**B**) Left panel: expression of CASC21 in CRC generated from RNA sequencing data from TCGA database. Right panel: qRT-PCR analysis of CASC21 expression in 80 pairs of CRC and corresponding adjacent normal tissues. (**C**) CASC21 expression in CRC cell lines (HT-29, HCT-116, SW-620, HCT-8 and SW-480) and normal colorectal epithelial cell FHC detected by qRT-PCR. (**D**) Kaplan-Meier survival analysis of CRC patients’ overall survival in cohort 1 based on CASC21 expression (n= 80, P= 0.004). (**E**) Kaplan-Meier survival analysis of CRC patients’ overall survival in cohort 2 based on CASC21 expression (n= 64, P= 0.004). All data represent mean ± SEM (n = 3-6). ^*^P < 0.05, ^**^P < 0.01 and ^***^P < 0.001.

First, we analyzed the transcriptome data of CRC in TCGA database and identified the abnormally expressed lncRNAs in CRC (Figure 1A). We found the expression levels of CASC21 was significantly increased in CRC tissues compared to adjacent tissues (Figure 1B, left panel). We also detected the expression of CASC21 in 80 pairs of CRC and adjacent tissues by qRT-PCR, as with the sequencing results, CASC21 was significantly elevated in tumor tissues (Figure 1B, right panel). To explore clinical relevance of CASC21 in CRC, we divided enrolled patients into two groups according to CASC21 expression. As shown in Table 1, statistical analysis demonstrated that CASC21 expression was correlated with tumor invasion depth (P = 0.003 in cohort 1, P = 0.003 in cohort 2) and TNM stage (P= 0.009 in cohort 1, P = 0.017 in cohort 2), but not related to age, gender, lymph node metastasis and distant metastasis. Besides, we examined the expression of CASC21 in a series of CRC cell lines and colorectal normal epithelial cells, and we found that it was significantly higher in CRC cell lines than in normal colorectal cells (Figure 1C). Furthermore, we evaluated the prognostic value of CASC21 in CRC. We divided the patients into two groups according to the CASC21 expression level and we found that the patients in the ‘high CASC21expression’ group had poor prognosis (Figure 1D, 1E).

**Table 1:** The clinic-pathological factors of CRC patients.

**Characteristics**	**Number of cases** ***(Cohort 1)***	**CASC21 expression**		**P value^a^**	**Number of cases** ***(Cohort 2)***	**CASC21 expression**		**P value ^a^**
		**Low (n= 40)**	**High (n= 40)**			**Low (n= 32)**	**High (n= 32)**	
**Age(year)**								
<60	32	14	18	0.361	28	13	15	0.614
≥ 60	48	26	22	36	19	17
**Gender**								
Female	44	20	24	0.369	27	16	11	0.206
Male	36	20	16	37	16	21
**Tumor invasion depth**								
T1-2	56	34	22	**0.003**	45	28	17	**0.003**
T3-4	24	6	18	19	4	15
**Lymph node metastasis**								
N0	58	32	26	0.133	46	25	21	0.266
N1+N2	22	8	14	18	7	11
**Distant metastasis**								
M0	65	35	30	0.152	52	27	25	0.522
M1+M2	15	5	10	12	5	7
**TNM stage**								
I+II	53	32	21	**0.009**	43	26	17	**0.017**
III+III	27	8	19	21	6	15

^**a**^ Statistical significant results (in bold).

### FOXP1 activates transcription of CASC21 in CRC

**Figure 2:**
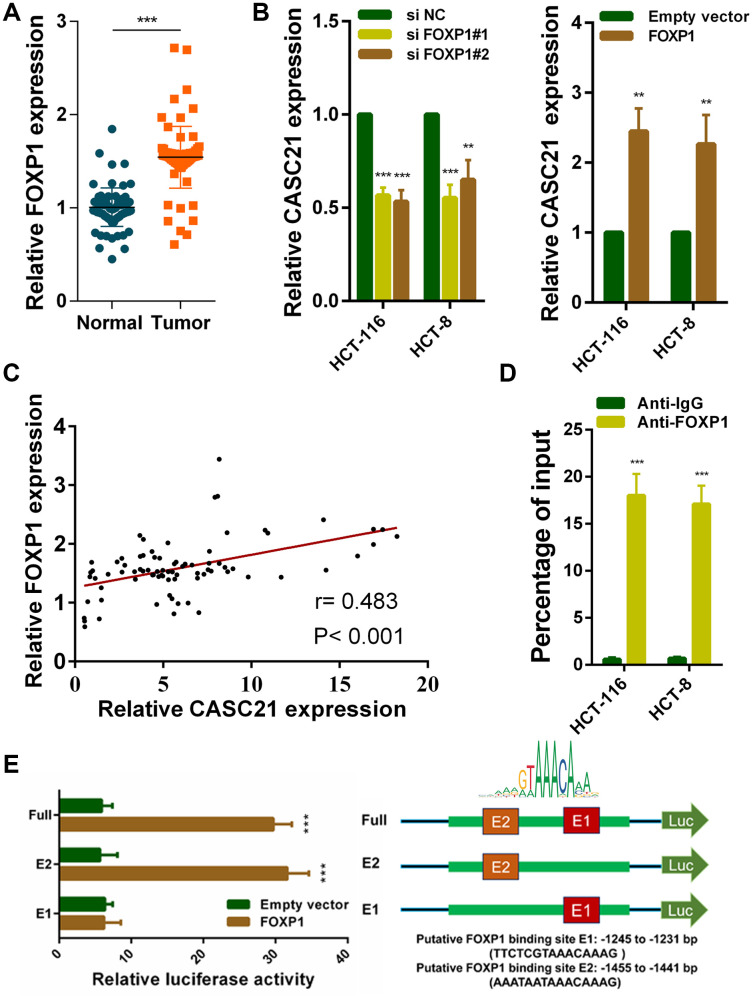
**FOXP1 induces CASC21 high expression in CRC.** (**A**) FOXP1 expression was detected by qRT-PCR in 80 pairs of CRC and corresponding adjacent normal tissues. (**B**) CASC21 expression was detected in HCT-116 and HCT-8 cells transfected with FOXP1 siRNAs or FOXP1 overexpression vector by qRT-PCR. (**C**) The correlation between FOXP1 and CASC21 expression analyzed in 80 paired CRC samples (n= 80, r= 0.483, P< 0.001). (**D**) ChIP assays were conducted to identify FOXP1 occupancy in the CASC21 promoter region. (**E**) Luciferase reporter assays were used to determine the FOXP1 binding sites on the CASC21 promoter region. All data represent mean ± SEM (n = 3-6). ^**^P < 0.01 and ^***^P < 0.001.

To explore potential factors that cause high expression of CASC21. We used the JASPER tool to predict transcription factors that might bind to the promoter region of CASC21 [22]. The results showed that FOXP1 has a higher probability of binding to it. Previous reports have shown that FOXP1 can induce the transcription of some lncRNAs. We examined the expression of FOXP1 in CRC tissues, and the results showed that FOXP1 expression was significantly elevated in CRC (Figure 2A). In addition, we detected FOXP1 expression in normal colorectal epithelial cells FHC and CRC cells. We found that FOXP1 expression in most CRC cells (except SW-480) was significantly higher than that in FHC (Supplementary Figure 1A). Survival analysis of FOXP1 revealed that high FOXP1 expression indicated a poor prognosis of CRC (Supplementary Figure 1B). We next silenced FOXP1 in HCT-116 cells and HCT-8 cells and found a decrease in the expression of CASC21. Similarly, overexpression of FOXP1 caused an increase in CASC21 expression (Figure 2B). We also found that FOXP1 expression was positively correlated with the expression of CASC21 in CRC tissues (Figure 2C). In addition, the results of the chip experiment indicated that FOXP1 can directly bind to the promoter region of CASC21 (Figure 2D). To further determine the specific region of FOXP1 binding to the CASC21 promoter, we performed a luciferase reporter assay. The results indicate that FOXP1 is mainly bound to the E2 region (-1455 to -1441 bp, AAATAATAAACAAAG), rather than to the E1 region (-1245 to -1231 bp, TTCTCGTAAACAAAG, Figure 2E).

### CASC21 can promote the growth of CRC cells *in vitro* and *in vivo*

**Figure 3:**
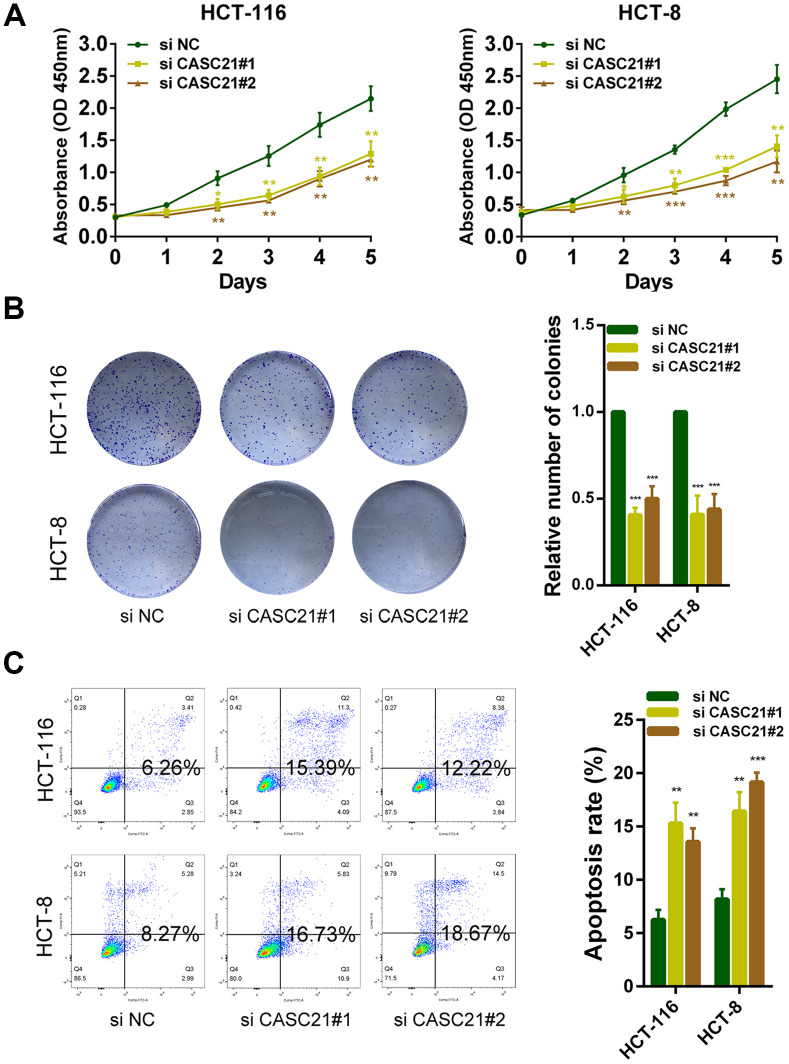
**CASC21 promotes CRC cells growth *in vitro*.** (**A**) CCK-8 assays of HCT-116 and HCT-8 cells transfected with CASC21 siRNAs. (**B**) HCT-116 and HCT-8 cells transfected with CASC21 siRNAs were seeded onto 6-well plates. The number of colonies was counted on the 14^th^ day after seeding. (**C**) Flow cytometric cell apoptosis assays used to assess the effect of CASC21 knockdown on cell apoptosis. All data represent mean ± SEM (n = 3-6). *P < 0.05, **P < 0.01 and ***P < 0.001.

**Figure 4:**
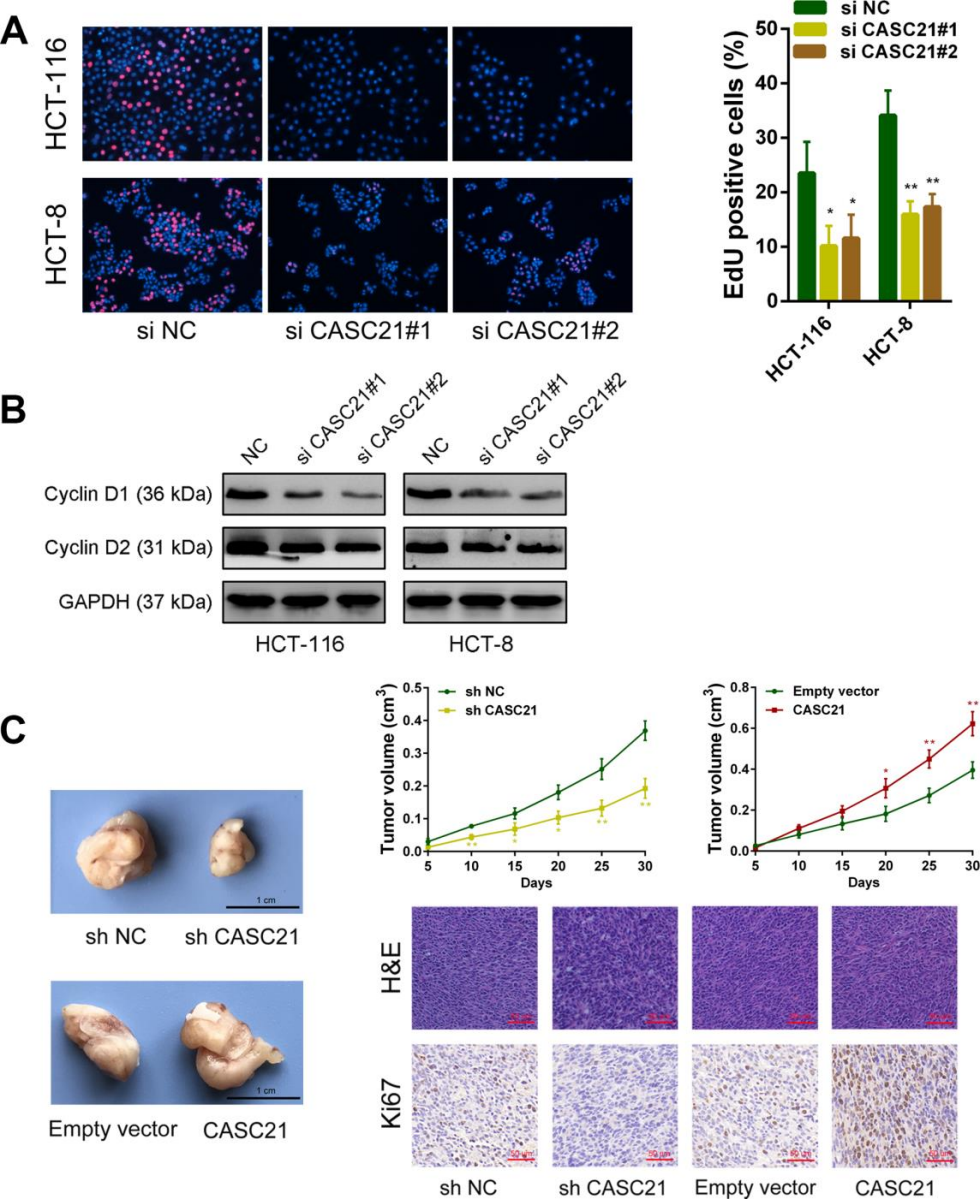
**CASC21 affects proliferation related proteins expression and promotes CRC growth *in vivo*.** (**A**) EdU assays were used to determine the cell proliferate ability of CASC21 siRNAs transfected cells. (**B**) Cell proliferation related proteins cyclin D1, and cyclin D2, were detected by western blot after CASC21 silencing. (**C**) Representative images of tumors formed in nude mice from negative control vector, sh-CASC21 vector and CASC21 overexpression groups, and the tumor volume growth curves after injections in different groups. Representative images for HE-staining and Ki67 immunostaining of tumor samples from different groups. All data represent mean ± SEM (n = 3-6). ^*^P < 0.05 and ^**^P < 0.01.

The results of CCK-8 experiments showed that the growth rates of HCT-116 and HCT-8 cells were significantly decreased after silencing CASC21 (Figure 3A). Similarly, after silencing CASC21, the numbers of clones of HCT-116 and HCT-8 cells were significantly lower than that of the control group (Figure 3B). Besides, cell apoptosis was detected by flow cytometry and the results indicated that the apoptosis rates of HCT-116 and HCT-8 cells were significantly increased when CASC21 was silenced (Figure 3C). In addition, the results of EdU assays displayed that the proportion of proliferative tumor cells decreased significantly after the knockdown of CASC21 (Figure 4A). Also, the results of western blot revealed that the protein levels of cyclin D1 and cyclin D2 in CRC cells were significantly reduced when CASC21 was silenced (Figure 4B). Next, we examined the effect of CASC21 on CRC cells *in vivo* through a nude mouse xenograft model. We found that the tumors formed by the CASC21 knockdown HCT-116 cells were significantly smaller than that of the control cells. Conversely, the tumors formed by the HCT-116 cells stably overexpressing CASC21 was significantly greater than that of the control group. The results of immunohistochemistry also demonstrated that the positive rate of Ki-67 in the CASC21 low expression group was lower than that in the control group. The positive rate of Ki-67 was higher in the CASC21 over-expressed group than that in the control group (Figure 4C).

### CASC21 acts as a competitive endogenous RNA which can sponge miR-539-5p in CRC

**Figure 5:**
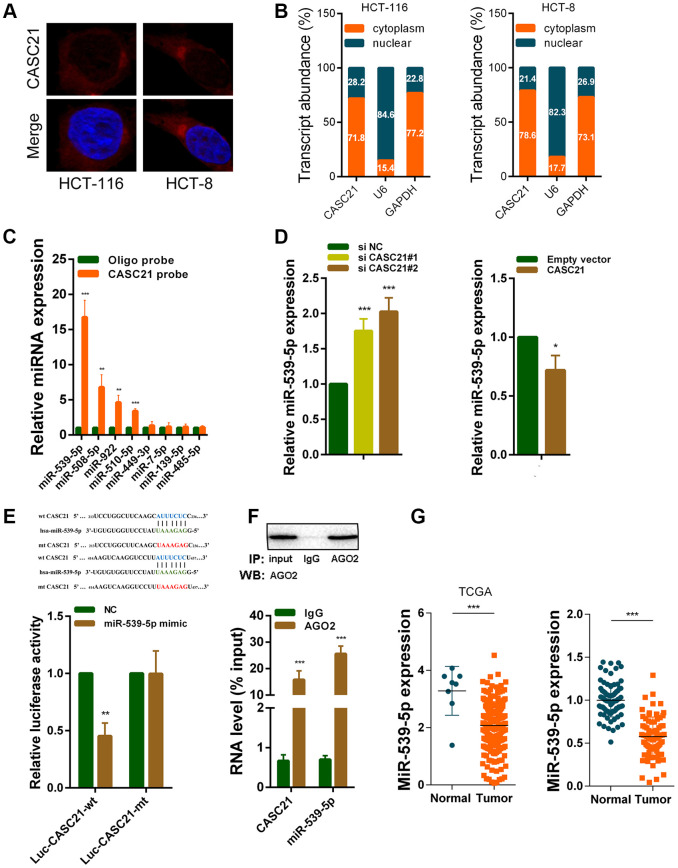
**CASC21 acts as a sponge for miR-539-5p in the cytoplasm.** (**A**) Representative FISH images showed the location of CASC21 in HCT-116 and HCT-8 cells (red). Nuclei were stained by DAPI (blue). (**B**) Relative CASC21 expression levels in the nuclear and cytoplasm fractions of HCT-116 and HCT-8 cells. Nuclear controls: U6, cytosolic controls: GAPDH. (**C**) The relative expression of candidate microRNAs which could potentially bind to CASC21 were quantified by qRT-PCR after the biotinylated-CASC21 pull-down assays in HCT-116 cells. (**D**) MiR-539-5p expression was detected in HCT-116 transfected with CASC21 siRNAs or CASC21 overexpression vector by qRT-PCR. (**E**) Dual luciferase reporter assays of wild type and mutant type (putative binding sites for miR-539-5p were mutated) CASC21 luciferase report vectors. Up panel, sequence alignment of miR-539-5p and their predicted binding sites (green) of CASC21. Predicted micorRNA target sequence (blue) in CASC21 (Luc-CASC21-wt) and positions of mutated nucleotides (red) in CASC21 (Luc-CASC21-mt). (**F**) RIP assays with an anti-Ago2 antibody to assess endogenous Ago2 binding RNAs, IgG was used as the negative control. The levels of CASC21 and miR-539-5p were determined by qRT-PCR and presented as fold enrichment in Ago2 relative to input. (**G**) Left panel: expression of miR-539-5p in CRC generated from RNA sequencing data from TCGA database. Right panel: qRT-PCR analysis of miR-539-5p expression in 80 pairs of CRC and corresponding adjacent normal tissues. All data represent mean ± SEM (n = 3-6). *P < 0.05, **P < 0.01 and ***P < 0.001.

We next explored the mechanism by which CASC21 plays an oncogene role in CRC. The results of RNA fluorescence in situ hybridization (FISH) experiments indicated that CASC21 was mainly distributed in the cytoplasm in HCT-116 and HCT-8 cells (Figure 5A). In addition, we conducted subcellular fractionation assays. The distribution of U6 and GAPDH proved that we have successfully isolated nucleus and cytoplasm and the results also showed that CASC21 was mostly distributed in the cytoplasm (Figure 5B). Many previous studies have shown that lncRNA located in the cytoplasm can act as a molecular sponge to adsorb miRNAs. We used the miRDB database and the MirTarget algorithm to search microRNA that theoretically bind to CASC21 [23, 24]. We found that a set of microRNAs (miR-539-5p, miR-508-5p, miR-922, miR-510-5p, miR-449-3p, miR-7-5p, miR-139-5p and miR-485-5p) were predicted to have a high probability of combining to CASC21. We next conducted RNA pull-down experiments with biotin-labeled CASC21 in HCT-116 cells. As shown in Figure 5C, miR-539-5p, miR-508-5p, miR-922 and miR-510-5p could be pulled down by CASC21. Among them, the miR-539-5p had the highest abundance binding to CASC21, so we chose it for further study. We found that expression of miR-539-5p was elevated following silencing of CASC21 in HCT-116 cells (Figure 5D, left panel). Conversely, overexpression of CASC21 in HCT-116 cells resulted in decreased expression of miR-539-5p (Figure 5D, right panel). Similar results were observed in HCT-8 cells and HT-29 cells (Supplementary Figure 2A, 2B). However, in FHC cells, overexpression of CASC21 caused a decrease in miR-539-5p, while silencing of CASC21 did not cause an increase in miR-539-5p expression (Supplementary Figure 2C). In addition, we constructed a wild-type luciferase reporter plasmid (wt-CASC21) and a mutant luciferase reporter plasmid (mt-CASC21) based on the predicted miR-539-5p and CASC21 binding sites. The results of the luciferase reporter assay indicated that miR-539-5p could bind to the target sequence on CASC21 (Figure 5E). We also performed RIP experiments with the AGO2 antibody, and the results showed that both miR-539-5p and CASC21 could directly interacted with endogenous AGO2 protein in HCT-116 cells (Figure 5F). By analyzing the TCGA database we found that the expression of miR-539-5p in CRC samples was significantly lower than that in adjacent tissues (Figure 5G, left panel). The same result was confirmed in the CRC samples of our cohort (Figure 5G, right panel). The result of survival analysis demonstrated that low miR-539-5p expression indicated a poor prognosis of CRC patient (Supplementary Figure 2D). In addition, correlation analysis revealed that the expression of miR-539-5p and CASC21 was negatively correlated in CRC tissues (Supplementary Figure 2E). We also examined the relationship between FOXP1 and miR-539-5p, and the results showed that silencing of FOXP1 caused a significant increase in the expression of miR-539-5p (Supplementary Figure 2F). The correlation analysis result indicated that FOXP1 expression was negatively correlated with the expression of miR-539-5p in CRC tissues (Supplementary Figure 2G). These above results demonstrated that CASC21 could work as a ceRNA to regulate miR-539-5p expression in CRC.

### CASC21 regulates CDK6 expression by adsorption of miR-539-5p in CRC

**Figure 6:**
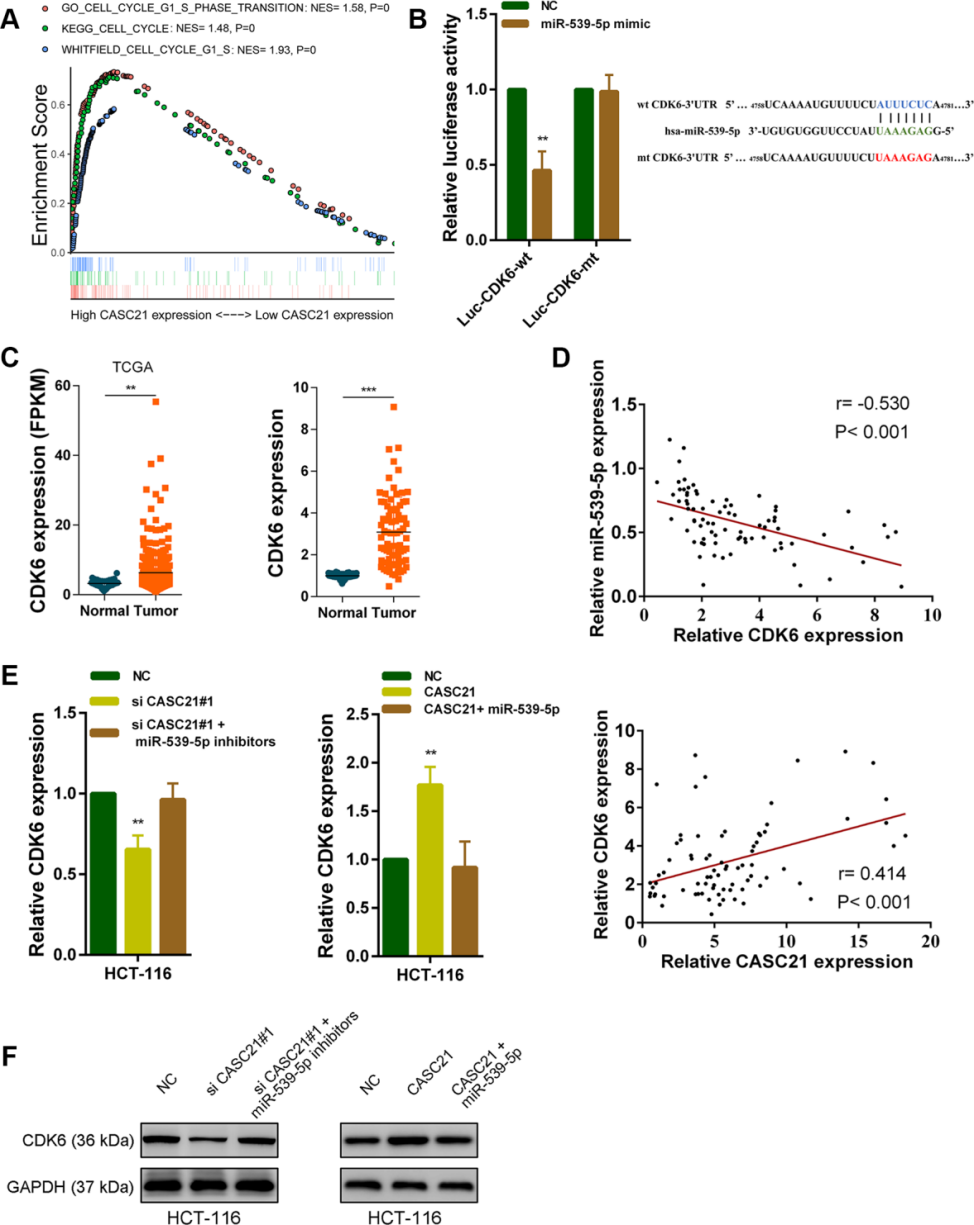
**CASC21 regulates CDK6 expression by sponging miR-539-5p in CRC.** (**A**) GSEA results were plotted to visualize the correlation between the expression of CASC21 and genes related to cell proliferation (KEGG_CELL_CYCLE, GO_CELL_CYCLE_G1_S_PHASE_TRANSITION and WHITFIELD_CELL_CYCLE_G1_S). (**B**) Dual luciferase reporter assays conducted with wild type and mutant type (putative binding sites for miR-539-5p were mutated) luciferase report vectors of CDK6 3’UTR. Right panel, sequence alignment of miR-539-5p and their predicted binding sites (green) of CDK6 3’UTR. Predicted micorRNA target sequence (blue) in CDK6 3’UTR (Luc-CDK6-wt) and positions of mutated nucleotides (red) in CDK6 3’UTR (Luc-CDK6-mt). (**C**) Left panel: expression of CDK6 in CRC generated from RNA sequencing data from TCGA database. Right panel: qRT-PCR analysis of CDK6 expression in 80 pairs of CRC and corresponding adjacent normal tissues. (**D**) Upper panel: The correlation between CDK6 and miR-539-5p in 80 paired CRC samples (n= 80, r= 0.-530, P< 0.001). Lower panel: The correlation between CDK6 and CASC21 in 80 paired CRC samples (n= 80, r= 0.414, P< 0.001). (**E**, **F**) CDK6 expression was detected by qRT-PCR or western blot in HCT-116 cells with indicated treatment. All data represent mean ± SEM (n = 3-6). **P < 0.01 and ***P < 0.001.

It is well known that mircroRNAs work by inhibiting the expression of target genes. We used the TargetScan and the miRDB database to search theoretical targets of miR-539-5p [24, 25]. Among potential target genes, CDK6 received high scores in both databases. Previous results have shown that CASC21 can affect CRC cell growth. Gene set enrichment analysis results manifested that CASC21 expression was positively correlated with the KEGG_CELL_CYCLE, GO_CELL_CYCLE_G1_S_PHASE_ TRANSITION and WHITFIELD_CELL_CYCLE_G1_S pathways (Figure 6A). These results suggested that CASC21 may influence cell cycle progression of CRC cells. So, we next explored whether CASC21 could regulate the expression of CDK6 by sponging miR-539-5p. We constructed a luciferase reporter plasmid based on the predicted binding sites of miR-539-5p and 3’-Untranslated Regions (3’-UTR) of CDK6 (wt CDK6-3’UTR), and constructed a mutant luciferase reporter plasmid with the binding sites mutated (mt CDK6-3’UTR). The results of the luciferase reporter assays showed that miR-539-5p could directly bind to the 3’-UTR of CDK6 (Figure 6B). Sequencing data from the CRC samples in the TCGA database indicated that CDK6 expression was significantly elevated in CRC (Figure 6C, left panel). The same result was observed in our CRC samples (Figure 6C, right panel). In addition, the expression of miR-539-5p and CDK6 was negatively correlated in CRC tissues, and the expression of CASC21 and CDK6 was positively correlated in CRC tissues (Figure 6D). Besides, we found that silencing CASC21 resulted in decreased expression of CDK6 in HCT-116 cells, but when silencing CASC21 while inhibiting miR-539-5p, reduced CDK6 would be rescued (Figure 6E, left panel). In line with our hypothesis, when CASC21 is overexpressed, the expression of CDK6 is elevated., and when both CASC21 and miR-539-5p was overexpressed, the tendency of CDK6 to increase will be eliminated (Figure 6E, right panel). The change of protein level was consistent with the change of RNA level in HCT-6 cells (Figure 6F). The same trend was also observed in HCT-8 and HT-29 cells (Supplementary Figure 3A, 3B). The result of survival analysis revealed that high CDK6 expression indicated a poor prognosis of CRC patient (Supplementary Figure 3C). In addition, we found that silencing FOXP1 caused a significant decrease in CDK6 expression (Supplementary Figure 3D). Correlation analysis showed that FOXP1 expression was positively correlated with CDK6 expression in CRC tissues (Supplementary Figure 3E).

### CASC21 promotes the growth of CRC cells through regulating the expression of CDK6 by sponging miR-539-5p

**Figure 7:**
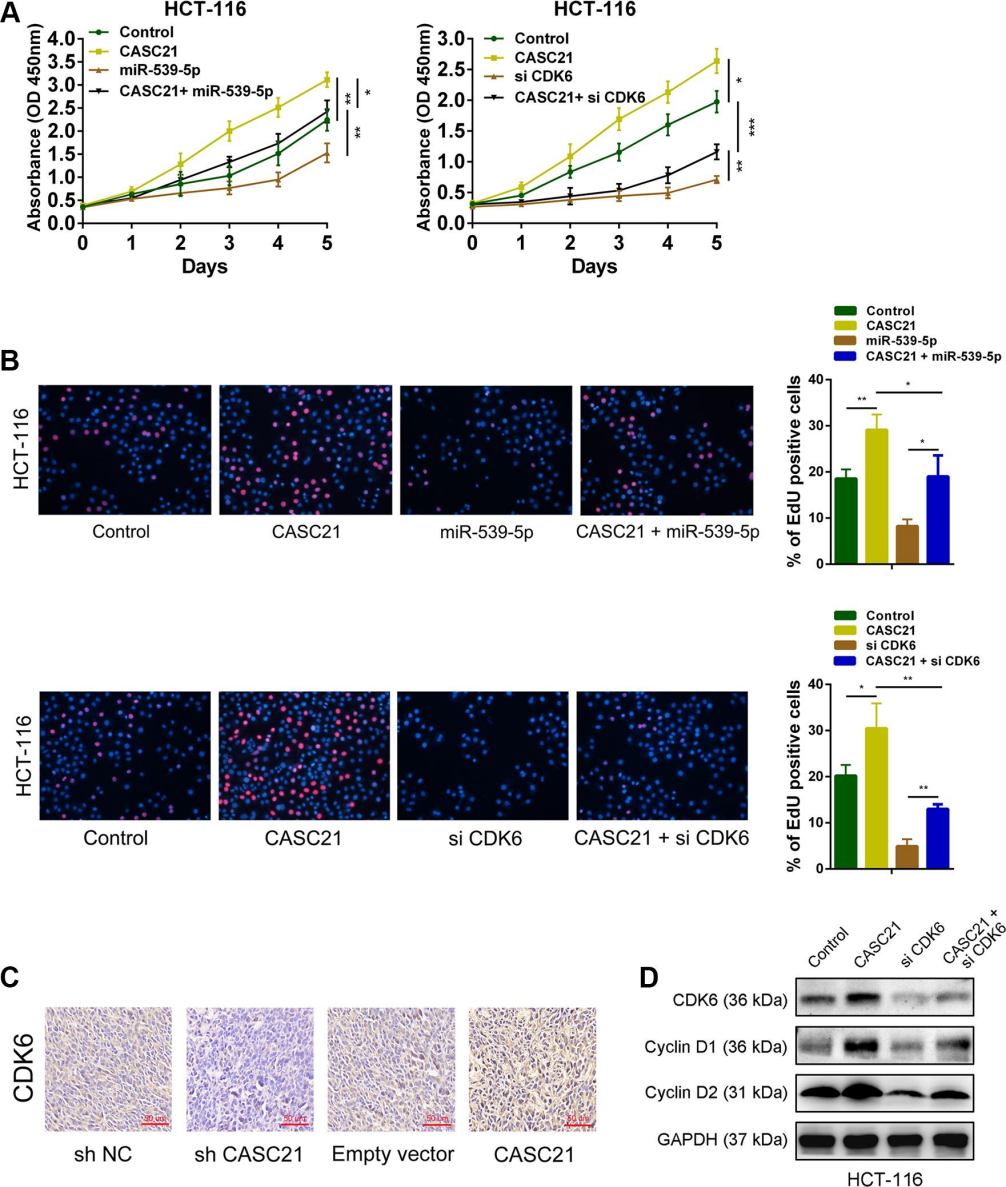
**CASC21 promotes CRC proliferation by regulating CDK6 expression.** (**A**) CCK-8 assays demonstrated that CASC21 promoted CRC cell growth, miR-539-5p overexpression or CDK6 knockdown could abolish growth promotion caused by CASC21. (**B**) EdU assays showed that miR-539-5p overexpression or CDK6 knockdown abolished the increased proliferation rates of HCT-116 cells caused by CASC21. (**C**) Representative images of CDK6 immunostaining of tumor samples from different groups. (**D**) Expression of CDK6, cyclin D1 and cyclin D2 was detected by western blot in HCT-116 cells with indicated treatment. All data represent mean ± SEM (n = 3-6). *P < 0.05, **P < 0.01 and ***P < 0.001.

The results of CCK-8 experiments showed that overexpression of CASC21 significantly promoted the growth of HCT-116 cells, while overexpression of miR-539-5p significantly inhibited the growth of HCT-116 cells. However, when overexpressed both CASC21 and miR-539-5p, the growth rate of HCT-116 cells was no more significantly different from that of the negative control group (Figure 7A, left panel). In addition, knocking down CDK6 will inhibit the growth rate of HCT-116, and when CASC21 was overexpressed while CDK6 was inhibited, the growth acceleration caused by the high expression of CASC21 will be significantly weakened (Figure 7A, right panel). The results of the EdU experiments indicated that overexpression of CASC21 promoted the proliferation of HCT-116 cells, and overexpression of miR-539-5p or knockdown of CDK6 attenuated the increase in proliferation rate caused by overexpression of CASC21 (Figure 7B). The results of immunohistochemistry revealed that overexpression of CASC21 resulted in an increase in the expression of CDK6 in mouse tumor-bearing tissues, and knockdown of CASC21 resulted in a decrease in the expression of CDK6 in tumor-bearing tissues (Figure 7C). The results of western blot showed that the expression of CDK6 in CRC cells was increased when CASC21 was overexpressed, and the expression of proteins level of cyclin D1 and cyclin D2 which were related to cell proliferation were also significantly increased. However, when over-expressing CASC21 while knocking down CDK6, the trend of increasing cyclin D1 and cyclin D2 will be significantly impaired (Figure 7D). These results indicated that CASC21 plays a role in promoting CRC proliferation by regulating the expression of miR-539-5p and CDK6 *in vitro* and *in vivo*.

## DISCUSSION

Our research has reported the expression pattern, biological roles and potential mechanisms of CASC21 in CRC. We first identified aberrantly expressed lncRNA in CRC tissues by analyzing RNA-Seq data. We found that CASC21 was abnormally elevated in CRC and confirmed the result by qRT-PCR. Combined with clinical data analysis, we found that the expression of CASC21 was associated with the prognosis of CRC patients, and high expression predicts a worse prognosis. In addition, the expression of CASC21 is also related to the tumor size and stage of CRC. These results suggest that CASC21 may play an important role in CRC. Besides, we found that the transcription factor FOXP1 could activate CASC21 transcription in CRC. Next, we demonstrated that CASC21 could promote CRC cells growth through a series of *in vitro* and *in vivo* experiments. Furthermore, we used luciferase reporter gene assays, RIP experiments to demonstrate that CASC21 could regulate the expression of CDK6 by adsorbing miR-539-5p, and this partly explained the mechanism by which CASC21 acted as an oncogene in CRC.

Many studies have shown that lncRNAs play important roles in the development and progression of tumors [26, 27]. Wang et al. reported that lncRNA EPIC1 could promote the cell-cycle progression of various tumor cells by interaction with MYC [28]. Bian et al. reported that lncRNA FEZF1-AS1 could promote CRC metastasis through activating STAT3 signaling [29]. Fu et al. reported that lncRNA HOTTIP could maintain pancreatic cancer stem cells properties through regulating HOXA9 [30]. CASC21 is a lncRNA located on chromosome 8q24. There are very few studies on it at present. Li et al. reported that CASC21 was a hotspot gene integrated by HPV in cervical cancer [31]. Interestingly, Zheng et al. also found that CASC21 played an oncogenic role in CRC. Their study demonstrated that CASC21 could promote CRC cells proliferation and metastasis through miR-7-5p/YAP1 axis [32]. We noticed that the CRC cell lines (HT-29, SW480) used in their research were different from ours (HCT-116, HCT-8). This indicates the heterogeneity of tumor cells and the complexity of molecular regulatory networks in cancer. Here, we revealed that CASC21 could regulate CDK6 expression through sponging miR-539-5p. LncRNAs in cytoplasm can act as a ceRNA to adsorb microRNAs and regulate the target genes of microRNAs, this is a classical way for lncRNA to function in cytoplasm [33]. MiR-539-5p was reported to act as a tumor suppressor in several cancers. Sun et al. demonstrated that miR-539-5p could inhibit nasopharyngeal carcinoma progression by targeting KLF12 [34]. Guo et al. revealed that miR-539-5p could inhibit glioma vasculogenic mimicry formation by decreasing expression of TWIST1 [35]. In this study, we found miR-539-5p was lowly expressed in CRC and could inhibit the proliferation of CRC cells by targeting CDK6. CDK6 is a well-known important cell cycle regulator which plays a critical role in tumor progression. It can combine with cyclinD to form a complex and promote the release of E2F family transcription factors, thereby promoting cell proliferation [36, 37]. We found that CASC21 regulated the proliferation of CRC cells in a miR-539-5p and CDK6-dependent manner. In CRC, various lncRNAs and microRNAs are reported to be involved in regulating CDK6 expression and our research provides new evidence for this phenomenon [19, 38, 39].

The expression of a lncRNA in tumor cells influenced by various factors, such as gene copy numbers, histone modification in promoter region and activation of transcription factors [40]. We found that CASC21 transcription could be induced by the FOXP1, a member of the Forkhead box transcription factors family which is involved in abroad range of roles, including carcinogenesis [41]. Wang et al. also reported that FOXP1 could activated transcription of the lncRNA CLRN1-AS1 in prolactinoma [42]. Remarkably, FOXP1 is identified to act as an oncogene, and its overexpression confers a poor prognosis in various types of cancers [43–46]. A recent study has demonstrated that FOXP1 participated in promoting the proliferation of CRC cells, and its high expression suggests poor prognosis of CRC patients [47]. We observed the same phenomenon in this study. We found that FOXP1 was highly expressed in CRC tissues and cells, and its high expression was associated with poor prognosis of CRC patients. FOXP1 could affect the proliferation of CRC by promoting CASC21 transcription. Our study provides new evidence for the role of FOXP1 in CRC.

In summary, our research proved that CASC21 played an oncogene role in CRC. It could increase the expression of CDK6 by sponging miR-539-5p, thereby promoting the proliferation of CRC cells. Our research provides a new theory for the molecular regulation of CRC and indicates CASC21 may be used as a potential target for the diagnosis or treatment of CRC.

## MATERIALS AND METHODS

### Tissue samples and clinical data collection

Two independent cohorts including 144 CRC patients were enrolled for this study. In cohort 1, 80 pairs of CRC tissues and their matched paracancerous tissue samples were collected from CRC patients who received radical surgery at the Jiangsu Province Hospital of Chinese Medicine, between March 2010 and March 2012. In cohort 2, 64 paired paraffin-embedded CRC specimens were collected from the Department of Pathology in Jiangsu Province Hospital of Chinese Medicine. All sample tissues were stored at liquid nitrogen container until RNA extraction. Written informed consents were obtained from all these patients, and this study was approved by the ethics committee on Human Research of the Jiangsu Province Hospital of Chinese Medicine, Nanjing University of Chinese Medicine. Clinical characteristics of these patients are listed in Table 1.

### Cell culture

The human CRC cell lines (HT-29, HCT-116, SW620, HCT-8 and SW480) and the normal colonic epithelial cells (FHC) were purchased from the ATCC. These cells were maintained in Dulbecco’s Modified Eagle Medium (DMEM, Gibco, USA) with 10% fetal bovine serum, 100 U/ml penicillin and 0.1 mg/ml streptomycin. For 96, 24, and 6-well plates, add 100, 500, and 2000 μl of culture medium to each well. For 6 cm or 10 cm dishes, add 6 ml or 10 ml culture medium, respectively. And they were incubated at 37°C in a humidified atmosphere with 5% CO2.

### RNA isolation and quantitative real-time PCR

Total RNA was extracted from tissues and cells using TRIzol reagent (Invitrogen, Carlsbad, CA, USA). Reverse transcription of lncRNA and mRNA was performed using a PrimeScriptTM RT Master Mix Kit (TaKaRa, Osaka, Japan). MiR-539-5p expression was detected by a Hairpin-it microRNA quantitation kit (Genepharma, China). Quantitative RT-PCR was performed using a standard protocol from the SYBR Green PCR kit (Toyobo, Osaka, Japan). The following primer sequences were used for qRT-PCR: for CASC21, GGTCAGAGTGGATCCAGAGG (forward) and GCCAACAGGAACCACATCTC (reverse); for CDK6, GCTGACCAGCAGTACGAATG (forward) and GCACACATCAAACAACCTGACC (reverse); for FOXP1, ATGATGCAAGAATCTGGGACTG (forward) and AGCTGGTTGTTTGTCATTCCTC (reverse); for GAPDH, GGAGCGAGATCCCTCCAAAAT (forward) and GGCTGTTGTCATACTTCTCAGG (reverse).

### Plasmid construction and cell transfection

The full-length complementary cDNAs of human CASC21 and FOXP1 were synthesized and cloned into the expression vector pcDNA3.1. The small hairpin RNA of CASC21 was synthesized and cloned into the pGLVH1/GFP/Puro vector (GeneCreate, China). CASC21, FOXP1 and CDK6 siRNAs were purchased from the Ambion (USA). Both miR-539-5p mimics and inhibitors were synthesized by the Ribobio (China). The plasmid vectors and siRNAs were transfected into CRC cells using Lipofectamine 3000 (Invitrogen, USA) according to the protocol.

### Chromatin immunoprecipitation assay

The ChIP assay was performed using the ChIP Assay Kit (Millipore, USA) following the manufacturer’s instructions. Briefly, 1x10^7^ of CRC cells were collected and cross-linked with 1% formaldehyde solution for 20 min. DNA fragments ranging from 200 to 500 bp were obtained by ultrasonication. Then the lysate was immunoprecipitated with anti-FOXP1 (#4402, Cell Signaling Technology, USA), or IgG antibodies. ChIP primer for the CASC21 promoter: GTGAGATGGTGAGGTGTGGA (forward) and CCCTTTGGCTCAAGGAACAC (reverse).

### RNA fluorescence in situ hybridization

FISH assays were performed using Fluorescent In Situ Hybridization Kit (RiboBio, China) according to the protocol. Cy3-labeled CASC21 probes were designed and synthesized by Ribobio (China).

### Subcellular fractionation location

The separation of the nuclear and cytosolic fractions was using the PARIS Kit (Invitrogen, USA) according to the manufacturer’s instructions.

### Western blot analysis

Cellular proteins were separated by SDS-polyacrylamide gel electrophoresis (4% stacking and 10% separating gels) and then transferred onto polyvinylidene fluoride membranes (Millipore, USA). The membranes were blocked with 5% skim milk for 1 h and then incubated with primary antibodies (anti-FOXP1 (#4402, Cell Signaling Technology, USA), anti-Cyclin D1(#55506, Cell Signaling Technology, USA), anti-Cyclin D2(#3741, Cell Signaling Technology, USA) anti- CDK6 (#13331, Cell Signaling Technology, USA) and anti-GAPDH(#5174, Cell Signaling Technology, USA)) overnight at 4 °C. After the membranes were incubated with secondary antibodies, they were subjected to immunoblot analysis using an ECL immunoblotting kit according to the manufacturer’s protocol.

### RNA immunoprecipitation assay

The RIP assay was performed using the Magna RNA immunoprecipitation Kit (Millipore, USA) following the the manual. Briefly, 1x10^7^ of CRC cells were lysed in RIP lysis buffer. Magnetic beads were pre-incubated with anti-AGO2 (#ab32381, Abcam, USA) or IgG antibody for 30 minutes at room temperature and the cell lysates was immunoprecipitated with beads for 6 hours at 4°C. After that, RNA was purified and detected by qRT-PCR.

### Luciferase reporter assay

For the CASC21 promoter luciferase reporter assay, different fragment sequences containing predicted FOXP1 binding sites were synthesized and cloned into the pGL3-basic firefly luciferase reporter (GeneCreat, China). The pRL-TK vector was employed as a control.

For microRNA target gene luciferase reporter assays, CASC21 sequences containing predicted miR-539-5p binding sites (or containing mutations in the predicted microRNA binding sites) were respectively synthesized and inserted into the pmirGLO luciferase vector (GeneCreat, China). 1x10^5^ of HCT-116 cells were seeded in 24-well plates for 24 h. Mimics of miR-539-5p were co-transfected with 10 μg wild type or mutated reporters. 24 hours after transfection, dual-luciferase reporter assay (Promega, USA) was performed to measure the relative luciferase activity. The same procedure was used to assess the binding effect between CDK6 3’UTR and miR-539-5p.

### Xenograft tumor formation

The xenografted tumor model was established to assess the effect of CASC21 *in vivo*. Briefly, 1×10^7^ HCT-116 cells (sh-NC, sh-CASC21, empty vector and CASC21 stably transfected) in 0.2 ml PBS were subcutaneously injected into BABL/c nude mice. The tumor volumes were measured every five days calculated with the following equation: V=0.5 × (length × width^2^). After one month later, the mice were sacrificed, and tumors were surgically dissected.

### Statistical analysis

All statistical analyses were performed using the SPSS 20.0. Survival curves were calculated using the Kaplan-Meier method and were analyzed using the log-rank test. For comparisons, one-way analyses of variance and two-tailed Student’s t-tests were performed, as appropriate. P< 0.05 was considered statistically significant.

A complete description of the methods, including CCK-8 assay and colony formation assay, 5-Ethynyl-20-deoxyuridine (EdU) incorporation assay, flow cytometry, RNA pull-down assay and Gene set enrichment analysis (GSEA) are available in the Supplementary materials and methods.

## Supplementary Material

Supplementary Materials

Supplementary Figures
