# Supplementary material for: CASC21, a FOXP1 induced long non-coding RNA, promotes colorectal cancer growth by regulating CDK6
Source: Aging (Albany NY). 2020 Jun 25;12(12):12086–106. doi: 10.18632/aging.103376 (PMC7343488; doi:10.18632/aging.103376)
Supplement: Supplementary Materials [file aging-12-103376-s001..pdf]

SUPPLEMENTARY MATERIAL

MATERIALS AND METHODS

CCK-8 assay and colony formation assay

For CCK-8 assay, HCT-116 and HCT-8 cells were seeded into 96-well plates at the density of 1×10^3 (cells/well), and the absorbance at 450nm was measured on days 1, 2, 3, 4 and 5 with 10 μl of CCK-8 solution treated. For cell colony formation assays, 24 hours after transfection, 500 HCT-116 or HCT-8 cells were incubated in 6-well plates at 37˚C, 5% CO_2. Two weeks later, the cells were stained with crystal violet (0.2%) for 30 minutes and the colony numbers were counted.

5-Ethynyl-20-deoxyuridine (EdU) incorporation assay

HCT-116 and HCT-8 cells were seeded at a density of 5×10^3 cells per well in 96-well plates and cultured overnight. The newly synthesized DNA of the cells was assessed by the EdU incorporation assay using a Cell-Light EdU DNA Cell Proliferation Kit (Ribobio, China), according to the manufacturer's instructions. The EdU incorporation rate was expressed as the ratio of EdU positive cells (red cells) to total Hoechst33342 positive cells (blue cells).

Flow cytometry

Flow cytometry cell apoptosis was analyzed using the Annexin V-FITC/ (PI) Apoptosis Detection Kit (BD, USA) according to the protocol. 1×10^6 of HCT-116 and HCT-8 cells were firstly trypsinized and washed with cold PBS, then they were stained with FITC and PI and then analyzed using FACScan (BD, USA). The cell apoptosis data were analyzed by Flowjo software (Tree Star, USA).

Gene set enrichment analysis

Gene set enrichment analysis (GSEA) was used to explore the pathways and gene sets associated with CASC21 in CRC. Gene expression profiles of CRC were downloaded from TCGA database. According to the CASC21 expression level, the samples were grouped as the high CASC21 group and low CASC21 group respectively. GSEA v3.0 was used to determine whether the members of the gene sets from the MSigDB database were randomly distributed at the top or bottom of the ranking. If most members of a gene set were positively or negatively related to CASC21, the set was associated with CASC21 expression.

RNA pull-down assay

CASC21 template DNA was transcribed in vitro with Biotin RNA Labeling Mix and T7 RNA polymerase (Roche, Switzerland) and purified with a RNeasy Mini Kit (Qiagen, USA) according to the manufacturer's instructions. RNA-bound beads were incubated with total cell lysates of HCT-116, and eluted RNA was purified and assessed by qRT-PCR.
