# Supplementary material for: CASC21, a FOXP1 induced long non-coding RNA, promotes colorectal cancer growth by regulating CDK6
Source: Aging (Albany NY). 2020 Jun 25;12(12):12086–106. doi: 10.18632/aging.103376 (PMC7343488; doi:10.18632/aging.103376)
Supplement: Supplementary Figures [file aging-12-103376-s002..pdf]

SUPPLEMENTARY FIGURES

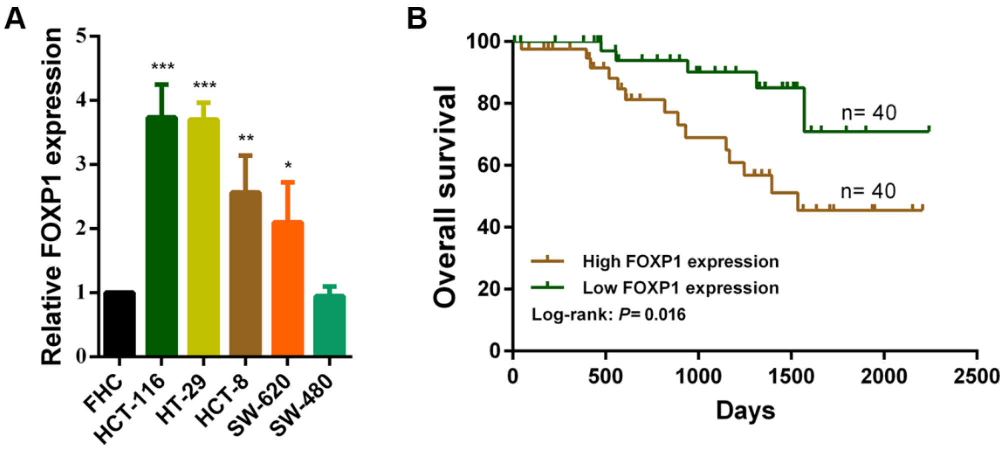

Supplementary Figure 1. FOXP1 is highly expressed in CRC cells and its high expression predicts poor prognosis. (**A**) FOXP1 expression in CRC cell lines (HT-29, HCT-116, SW-620, HCT-8 and SW-480) and normal colorectal epithelial cell FHC detected by qRT-PCR. (**B**) Kaplan-Meier survival analysis of CRC patients' overall survival in cohort 1 based on FOXP1 expression (n= 80, P= 0.016).

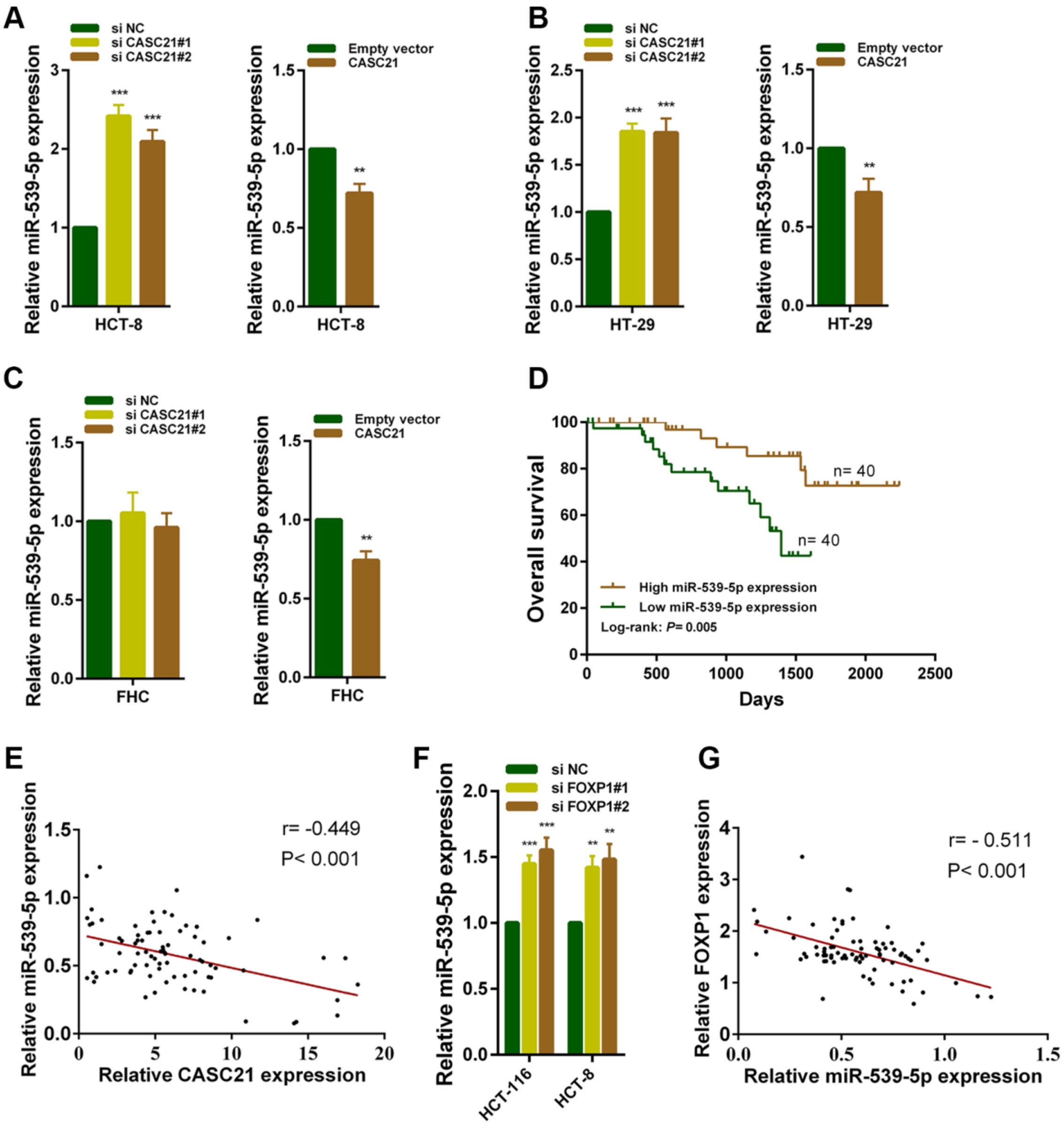

Supplementary Figure 2. CASC21 functioned as a molecular sponge for miR-539-5p. (**A**) MiR-539-5p expression was detected in HCT-8 cells transfected with CASC21 siRNAs or CASC21 overexpression vector by qRT-PCR. (**B**) MiR-539-5p expression was detected in HT-29 cells transfected with CASC21 siRNAs or CASC21 overexpression vector by qRT-PCR. (**C**) MiR-539-5p expression was detected in FHC cells transfected with CASC21 siRNAs or CASC21 overexpression vector by qRT-PCR. (**D**) Kaplan-Meier survival analysis of CRC patients' overall survival in cohort 1 based on miR-539-5p expression (n= 80, P= 0.005). (**E**) The correlation between CASC21 and miR-539-5p was analyzed in 80 paired CRC samples (n= 80, r= 0.-449, P< 0.001). (**F**) MiR-539-5p expression was detected in HCT-116 and HCT-8 cells transfected with FOXP1 siRNAs by qRT-PCR. (**G**) The correlation between FOXP1 and miR-539-5p was analyzed in 80 paired CRC samples (n= 80, r= 0.-511, P< 0.001).

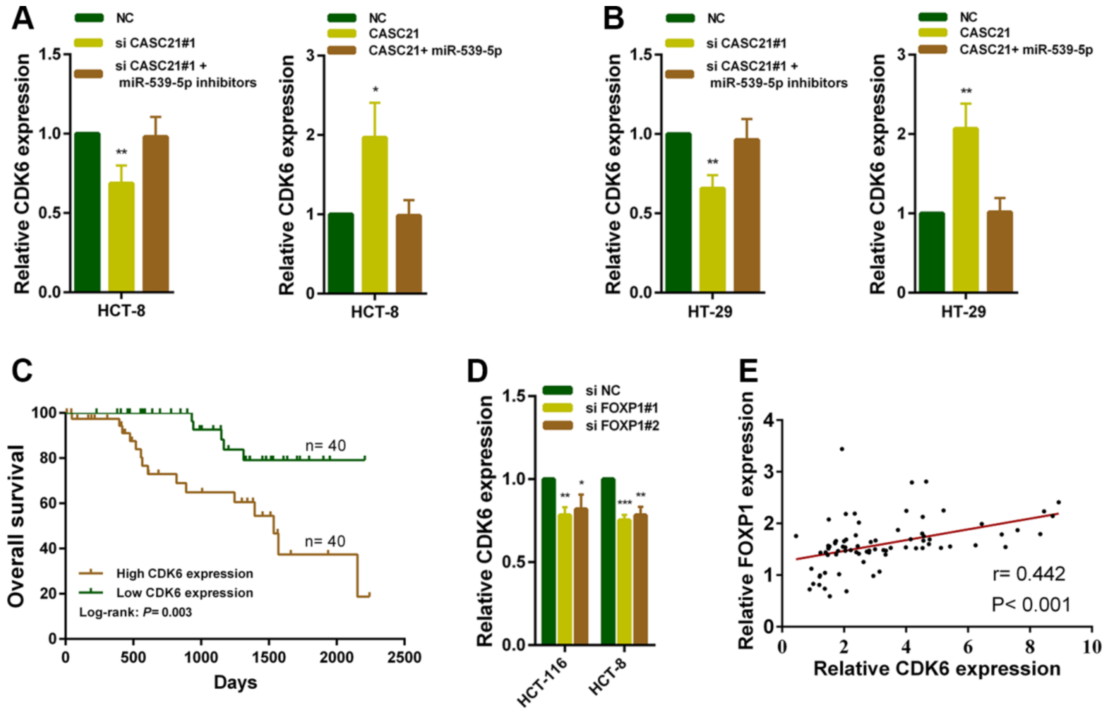

Supplementary Figure 3. CASC21 increased CDK6 expression by absorbing miR-539-5p in CRC. (**A**, **B**) CDK6 expression was detected by qRT-PCR in HCT-8 or HT-29 cells with indicated treatment. (**C**) Kaplan-Meier survival analysis of CRC patients' overall survival in cohort 1 based on CDK6 expression (n= 80, P= 0.003). (**D**) CDK6 expression was detected in HCT-116 and HCT-8 cells transfected with FOXP1 siRNAs by qRT-PCR. (**E**) The correlation between CDK6 and FOXP1 was analyzed in 80 paired CRC samples (n= 80, r= 0.442, P< 0.001).
